# Delay in discharge and its impact on unnecessary hospital bed occupancy

**DOI:** 10.1186/1472-6963-12-410

**Published:** 2012-11-20

**Authors:** Muhammad Umair Majeed, Dean Thomas Williams, Rachel Pollock, Farhat Amir, Martin Liam, Keen S Foong, Chris J Whitaker

**Affiliations:** 1 Department of Vascular Surgery, Ysbyty Gwynedd Hospital, Bangor, LL57 2PW, UK; 2 School of Medical Sciences, Bangor University, Bangor, LL57 2UW, UK

**Keywords:** Surgery, Elderly, Length of stay, Bed occupancy, Community care

## Abstract

**Background:**

Elderly patients are potentially more vulnerable to prolonged hospital stay as they frequently require additional resources to facilitate their discharge. In an acute hospital setting, we aimed to quantify and compare length of stay (LOS) for all patients over and under the age of 65, and identify the number and cause of days lost under the care of a single surgical unit.

**Methods:**

Over a 4 month period from January to April 2010, data on the management and source of potential delay was collected daily on consecutive patients admitted and discharged under the care of one consultant surgeon at a district general hospital. Statistical analysis was then performed with particular focus on actual delays affecting elderly patients.

**Results:**

A total of 99 complete inpatients episodes were recorded. There were 30 elective and 69 acute admissions. 10 (33%) elective vs. 42 (61%) acute patients encountered delays, losing 39 and 232 days respectively (χ^2^ [1, N = 99] = 6.36, p = .012). 23 of a total 39 elderly patients admitted acutely required specialist care of the elderly opinion and placement in community hospitals resulting in delays of 188 days. vs. 36 days for the 16 discharged home and 8 days for 30 patients under 65 (χ^2^ (2, N = 69) = 26.54, p = <.001).

**Conclusions:**

Elderly patients experiencing acute surgical admission and discharge to community hospitals had prolonged LOS due to significant delays associated with care of the elderly provision. The financial considerations behind bed capacity in primary and secondary care and the provision of care of elderly services need to be balanced against unnecessary occupancy of acute hospital beds with its associated health and economic implications.

## Background

Delayed discharge or ’bed blocking’ are terms used to describe the inappropriate occupancy of hospital beds. Delay in discharging surgical patients from hospital is a long-standing and common problem [1]. Delayed discharges have an impact on hospitals’ ability to cut waiting lists and deliver healthcare effectively and efficiently. In acute care hospitals, prolonged length of stay (LOS) not only increases cost, but is also associated with increased rates of complications [2]. LOS is being used to analyse surgical performance as part of efficiency drives and financial pressures have emphasised the importance of expeditious hospital discharge. Identification of the barriers to timely discharge may help direct efforts towards reducing unnecessary hospital stay.

Length of stay for general surgical patients is influenced by many variables. These variables include direct and indirect medical influences such as waiting for investigations and making home arrangements [3].

Despite a multidisciplinary approach to patient healthcare and discharge planning, it was anecdotally noted that surgical patients, particularly the elderly, under the care of one surgical team, were experiencing prolonged stays for reasons which were largely avoidable and not directly related to surgical activity.

With the aim of quantifying this apparent effect, a study was performed to prospectively collect data on LOS and identify the number and cause of days lost for patients under the care of a single surgical unit.

## Method

Over a 4 month period from January to April 2010, data was prospectively and consecutively collected for all emergency and elective patients admitted and discharged under the care of one Consultant vascular and general surgeon. Patients directly admitted in an unplanned manner or transferred following emergency admission under another team were categorised as acute admissions. All other admissions were deemed elective. Details collected included date of admission, indications for admission, the date and time of procedures requested (diagnostic/therapeutic), referrals made, date of planned and actual discharge, and LOS.

Patients undergoing procedures, receiving consultations or discharged within 24 hours of the documented time and date of the request or event, were deemed not to have encountered delay. All day case patients (admitted and discharged on same day) and patients who were still in hospital at the end of the 4 month period were excluded from the study. To facilitate accurate data collection on a busy surgical firm, an integral and dedicated team member, the foundation house officer, entered data daily for every patient onto a flow chart. The duration of the study and thus the number of patients included was based solely on the duration of attachment of the foundation house officer to the firm.

Delays were divided into those that occurred pre- or post-discharge planning. Pre discharge planning involves medical and nursing staff collectively predicting a date of discharge for each patient. In our unit, predicted date of discharge (PDD) is used to facilitate discharge arrangements for patients whom we anticipate on admission, are likely to require a hospital stay of over five days duration. Patients identified as having post-discharge planning delay were those who stayed in hospital beyond the date of discharge agreed by the Consultant and nursing staff.

Delays prior to discharge planning due to diagnostic/therapeutic procedures, waiting for consultant opinion (delay following referral for specialist opinion) or errors made by the team (e.g. failure to request in a timely manner a certain diagnostic test) were initially considered potential delays. The data on potential delays was then further assessed on an individual patient basis by the Consultant and his team at point of discharge to determine whether the delays encountered would have made any impact on the LOS. Potential delays that directly influenced LOS were deemed definite and included in the final analysis. Potential delays regarded as having no impact on LOS were excluded.

Delays due to care of the elderly (COTE) referral for elderly patients aged 65 years and over who required assessment as part of the discharge arrangements and continuing care were included in the analysis when the PDD was breached. COTE referrals are required when patients require discharge to community hospitals, as COTE teams are responsible for the continued care of in-patients in those hospitals. All such referrals were done as part of multidisciplinary approach and are sent immediately following the decision on PDD. Data was analysed for total LOS, median LOS, days lost due to delays and factors causing delays. Statistical analysis was performed using Student’s t-test for parametric data and Mann–Whitney U test for non-parametric LOS data on SPSS v.15 (Chic.,Ill.).

## Results

Over a 4 month period, a total of 99 complete inpatients episodes were recorded. There were 30 elective and 69 acute patients. Ten (33%) elective and 42 (61%) acute patients encountered delay. The proportion of patients with delays was significantly different in the acute and elective groups (χ^2^ [1, N = 99] = 6.36, p = .012).

The total bed occupancy was 1408 days and median LOS 8 days (range 1–86 days). Of 146 days potentially lost due to delays, 82 were deemed to have impacted unnecessarily on LOS before the discharge planning occurred (Table 1). There were 189 days lost after discharge date was set. Delays therefore accounted for 271 days (19%) of the TBO.

**Table 1:** The contribution of each cause of delay in the acute and elective admissions groups

			**Elective (N = 30) (30%) 39 days**	**Acute (N = 69) (70%) 232 days**	**Mann–Whitney U test**
Age		Median	67.0	64.5	
		Mean	63.91	63.47	
		(SD)	(19.45)	(9.25)	
Total delay (days)		Sum	39(14.1%)	232(85.6%)	p = 0.043
		Median	0	1	
		Mean	1.30	3.36	
		(SD)	(2.09)	(5.79)	
	Discharge arrangements	Sum	1(0.37%)	101(37.2%)	p < 0.001
		Median	0	0	
		Mean	0.03	1.460	
		(SD)	(3.85)	(0.18)	
	COTE	Sum	4(1.5%)	88(32.4%)	p = 0.021
		Median	0	0	
		Mean	0.03	1.28	
		(SD)	(3.38)	(0.18)	
	Others	Sum	18(6.6%)	4(1.4%)	p = 0.090
		Median	0	0	
		Mean	0.73	0.06	
		(SD)	(0.29)	(1.91)	
	Investigations & therapies	Sum	11(4%)	39(14.3%)	p = 0.451
		Median	0	0	
		Mean	0.37	0.57	
		(SD)	(1.35)	(1.03)	
	Team errors	Sum	5(1.8%)	0(0%)	p = 0.031
		Median	0	0	
		Mean	0.17	0	
		(SD)	(0.65)	(0)	

Abbreviations.

COTE: Care Of The Elderly consultant referral.

SD: Standard Deviation.

**Figure 1:**
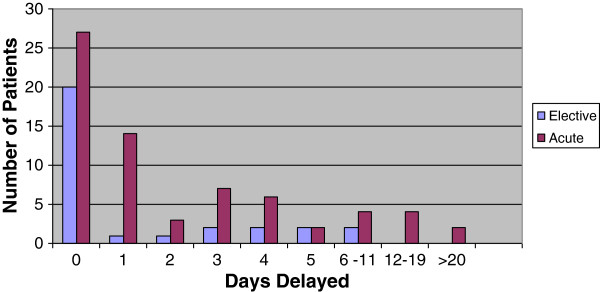
Distribution of delays for all elective and acute admissions.

The mean length of stay for elective patients was 1.30 days vs. 3.36 days for acute admissions. The number of days delayed showed a positive or right skewed distribution with median equalling 0 for the majority of measurements.(Figure 1) Using a Mann–Whitney U test, there were significant differences in days lost due to delay between the elective and acute groups, mean (S.D.) 3.36 (5.79) vs. 1.30 (2.09) respectively, p = .043. (Table 1).

Employing an independent samples t test, the age of patients in the elective and acute groups were not significantly different, median 64.5 and 67.0 years, respectively (t_97_ = 0.12, p = .905).

### Causes of delay

#### Acute vs. elective admissions

Of 39 days delay in the elective group, only one day was related to discharge arrangements and the four referrals to COTE. This compares with 101 (37.2%) and 88 (32.4%) days lost due to discharge arrangements and COTE referral in the acute group (Mann–Whitney U-test p < .001and p = .021 respectively) (Table 1). Delays incurred from referral to other specialties and waiting for tests/therapies were not significantly different in the elective vs. acute groups, 18(6.6%) vs.4 (1.4%) days and 11(4%) vs. 39(14.3%) days respectively, p = .090 and p = .451 respectively. 5(1.8%) days were lost due to team errors, all in the elective group.

The total 271 days delay affected 52 patients. 47 patients did not encounter delay. Interestingly 11 elderly patients, all acute admissions requiring COTE referrals accounted for 139 days.

#### Age associated delays

The age of the 30 admissions in the elective group were equally divided between those under and over 65 years. Delays in the under 65 group totalled four days. Four (26%) of the 15 elderly patients were referred to COTE compared to 23 (59%) of the total 39 elderly patients referred in the acute group.

In the acute group, 30 patients under 65 years accounted for a total eight (3.5%) days delay. Of the total 39 elderly patients, the 23 referred to COTE incurred 188 (81%) days delay vs. 36 (15.5%) days for the 16 remaining (Table 2). Delays surrounding discharge arrangements in those referred to COTE accounted for 92 (39.6%) days vs. 6 (2.5%) for those not requiring COTE (χ^2^ 27.39 [2] p < .001).

**Table 2:** Number of days delay associated with patient age and care of the elderly referral for community hospital placement in the acute admissions group

**Age**		**Discharge arrang**	**Care of elderly ref**	**Other specialist ref**	**Invest & therapy**	**Team errors**	**TOTAL**
Age less than 65	Mean days	0.10	0.00	0.07	0.10	0.00	0.27
N = 30	Std. Deviation	0.305	0.000	0.365	0.548	0.000	0.691
(43.4%)	Sum	3	0	2	3	0	**8(3.5%)**
Age 65 & over	Mean days	0.38	0.00	0.13	1.75	0.00	2.25
No COTE referral	Std. Deviation	0.806	0.000	0.342	1.880	0.000	1.528
N = 16	Sum	6	0	2	28	0	**36(15.5%)**
(23.1%)							
Age 65 & over	Mean days	4.00	3.83	0.00	0.35	0.00	8.17
COTE referral	Std. Deviation	5.916	5.015	0.000	1.191	0.000	7.947
N = 23	Sum	92	88	0	8	0	**188(81%)**
(33.3%)							
Total	Mean days	1.46	1.28	0.06	0.57	0.00	3.36
N = 69	Std. Deviation	3.845	3.382	0.291	1.345	0.000	5.788
	Sum	101	88	4	39	0	**232(85%)**

## Discussion

This study attempted to measure the number and causes of unnecessary, additional inpatient days for surgical patients and not whether they were inappropriate admissions.

The definition and therefore measurement of inappropriate hospital admissions and unnecessary inpatient stay (day of care criteria), has been much studied using the original or variations on the Appropriateness Evaluation Protocol (AEP) [4]. Although the AEP has helped in objectively measuring inappropriate hospital stay, it has many limitations [5]. Other studies have identified limitations surrounding the measurement of delayed discharge and its causes [6]. Many studies have employed retrospective analyses following discharge with inherent limitations in data quality, often failing to accurately quantify the influence of various sources of delay on overall bed occupancy for each patient. This study was a detailed, prospective study that attempted to analyse not only the source of delay from admission to discharge, but also the impact of the delays on the overall bed occupancy.

The study endeavoured to accurately quantify the unnecessary time spent by patients in hospital by excluding delays that did not ultimately influence the LOS, e.g. delays waiting for a scan whilst receiving treatment for an infection may not influence LOS.

LOS is widely used as an indicator of hospital performance because it is an objective outcome measure of resource utilization.

This study is limited in that it represents a snapshot and may not accurately reflect activity over one year.

We have demonstrated that almost 20% of the total LOS for our unit over this period was not related to surgical activity. Delays caused by the surgical team itself amounted to only 5 days (2% of total delays) in 4 months.

To illustrate this in terms of bed utilisation, if for example we assume an average LOS for elective patients on a surgical ward is 7 days, then the 266 days lost due to avoidable non-surgical delays equates to admitting and discharging a further 38 patients during this 4 month period, or greater than 100 patients over a year.

The study has further highlighted that although there were delays waiting for tests and procedures, the major influences were waiting for consultant opinions and failure of discharge arrangements, together accounting for 80% of prolonged LOS.

The two major contributors to the discharge delays were intimately related in the elderly because of the requirement of referral to COTE consultants to facilitate rehabilitation or continuation of care outside the acute hospital setting.

The surgical team in this study cares for a high proportion of more dependent, elderly individuals and is actively involved with many healthcare workers in discharge planning and predicting discharge dates.

Patients with peripheral vascular disease will frequently have several co-morbidities including chronic respiratory and heart problems, often accompanied by limited mobility. As a consequence, they frequently require intensive multidisciplinary input that can highlight deficiencies in healthcare that lead to delays in discharge. Several studies have identified problems in communication among colleagues and different disciplines involved in patient care that influenced LOS and care [7,8].

The joint assessment of readiness for discharge and PDD avoids variation in opinions between staff on the appropriateness of discharge highlighted as a weakness in other studies [9]. PDD aims to reduce LOS by initiating discharge planning activity as soon as it is possible, allowing time to accommodate and co-ordinate the arrangements. The predictable and timely discharge of patients employing PDD can occur in a safe manner [10].

The dependent and complex nature of the patients on our unit has meant that daily consultant rounds, the promotion of multi-disciplinary input and active engagement in treatment and discharge planning has been the standard for many years. Where it is evident that the patient will require additional allocation of resources for discharge to the community, discharge planning is started as soon as possible.

Delayed discharge and prolonged LOS not only has economic implications but also affects patients’ functional status. Previous studies have suggested that prolonged LOS is associated with significant complications including nosocomial infections, poor mobility, pressure sores, deep vein thrombosis and de-conditioning for living independently, thus worsening patients’ quality of life [11].

Initiatives to reduce waiting times for inpatient imaging and treatments have previously been introduced. We have identified that the major influences causing delay during this study relate to consultant opinion and waiting for arrangements in the community, particularly for elderly patients. Waiting times for tests and procedures account for less than 20% of prolonged LOS.

At our institution, care of the elderly clinicians have been over-stretched and frequently been unable to assess patients in a timely manner. Further, the number of beds in community hospitals has been in decline for several years. Frequently, patients accepted by COTE clinicians cannot be discharged because of unavailability of beds in community hospitals. Currently, an initiative has been introduced employing a senior nurse to guide patient discharge arrangements. This study used the PDD as the point after which delays due to COTE opinion were included. Therefore, although this incentive may have some influence the time waiting for an opinion regarding discharge, it is likely to have little influence on delays due to community provision.

Discharge delays due to lack of availability of post-discharge facilities and waiting for consultant opinions, tests and procedures, have been identified previously [12,13]. This study has attempted to accurately quantify delays and their causes by adjusting for each patient, potential delays that did not lead to a prolonged LOS.

The study has confirmed that the same issues persist, but highlights that it is elderly patients admitted acutely and requiring on-going community care that are greatly affected [14].

There is growing demand for emergency inpatient beds whilst in the current financial climate there is a drive to cut bed numbers. Despite recommendations on improving capacity in community hospitals to compensate for loss of acute beds and reduce LOS, they continue to decline [15,16].

## Conclusions

The utility of acute hospital beds is therefore of particular importance at this time. Our unit is embarking on an initiative to facilitate patient discharge, decrease the demands on community beds and reduce the frequency of re-admission by employing out-reach nurses to provide continuity of care between the hospital and community by visiting and managing patients in their homes and community hospitals.

An increasingly elderly population will place additional demands on healthcare provision. The financial considerations behind hospital bed planning in primary and secondary care and care of the elderly provision need to be balanced against the unnecessary prolonged occupancy of acute care beds and its associated health and financial implications.

## Competing interests

The author declares that they have no competing interest.

## Authors’ contributions

MUM and DTW reviewed the literature and drafted the manuscript. DTW reviewed the manuscript and supervised the conception and design of the study., RP, FA, ML and KSF collected and analyzed the data. CJW did the statistical analysis of data. All authors read and approved the final manuscript.

## Pre-publication history

The pre-publication history for this paper can be accessed here:

http://www.biomedcentral.com/1472-6963/12/410/prepub
